# A Solid-State NMR Investigation of MQ Silicone Copolymers

**DOI:** 10.1007/s00723-013-0456-8

**Published:** 2013-05-31

**Authors:** Sergey G. Vasil’ev, Vitaly I. Volkov, Elena A. Tatarinova, Aziz M. Muzafarov

**Affiliations:** 1 Institute of Problems of Chemical Physics, Academician Semenov Avenue 1, Chernogolovka, 142432 Russian Federation; 2 Institute of Synthetic Polymeric Materials, Profsoyuznaya Street 70, Moscow, 117393 Russian Federation

## Abstract

The structure of MQ copolymers of the general chemical formula [(CH_3_)_3_SiO_0.5_]_m_ [SiO_2_]_n_ was characterized by means of solid-state magic angle spinning (MAS) nuclear magnetic resonance (NMR) spectroscopy. The MQ copolymers are highly branched polycyclic compounds (densely cross-linked nanosized networks). MQ copolymers were prepared by hydrolytic polycondensation in active medium. ^29^Si NMR spectra were obtained by single pulse excitation (or direct polarization, DP) and cross-polarization (CP) ^29^Si{^1^H} techniques in concert with MAS. It was shown that material consist of monofunctional M (≡SiO *Si* (CH_3_)_3_) and two types of tetrafunctional Q units: Q^4^ ((≡SiO)_4_ *Si*) and Q^3^ ((≡SiO)_3_ *Si*OH). Spin–lattice relaxation times *T*_1_ measurements of ^29^Si nuclei and analysis of ^29^Si{^1^H} variable contact time signal intensities allowed us to obtain quantitative data on the relative content of different sites in copolymers. These investigations indicate that MQ copolymers represent dense structure with core and shell.

## Introduction

Organic–inorganic hybrid nanomaterials have attracted considerable interest owing to its unique optical, magnetical, and electronic properties of particles at the nanoscale [1–5]. The hybrid nanoparticles commonly consist of the rigid inorganic core surrounded by the soft polymer shell [2, 3]. The typical examples for the inner part are SiO_2_, Fe_2_O_3_ and some other transitional metal oxides or small metal clusters such as Au or Si particles. The shell is typically based on polymers such as polystyrene or polyacrylates [2, 4, 6–8]. Frequently, the interest of such materials is focused on the properties of the core material, such as optical or magnetic properties whereas shell provides good solubility or ability to be dispersed in polymer matrix preventing the aggregation of particles. The addition of inorganic nanosized fillers reveals promising strategy for fabrication of high performance polymer-based materials through the combination, in the same system, of the peculiar properties of organic and inorganic components [3, 4].

**Fig. 1:**
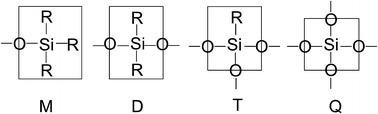
Widely accepted abbreviations used for silicone groups

Silicones are compounds of silicon that possesses at least one silicon–carbon bond and have a siloxane linkage. The designation M, D, T and Q, respectively, are used for mono-, di-, tri-, and quaternary coordination of oxygen around silicon in silicones. This common “shorthand notation” for silicones is depicted in Fig. 1, in which R is usually a methyl (–CH_3_) group. MQ copolymers are well-known hybrid material [11]. These siloxane copolymers comprise tetrafunctional inorganic Q units and organic monofunctional M trimethylsiloxane units. Although MQ copolymers are widely used in practice over a long period of time their detailed structures are not clear owing to the diversity of feasible variants corresponding to the same chemical composition.

The hydrolytic polycondensation of functional silicon derivatives is one of the most important methods for synthesis of polysiloxanes with the various compositions ranging from the organocyclosiloxanes to the high-molecular-weight polymers of linear, cyclic-linear and branched structure. Particular diversity of the hydrolytic polycondensation is the widely applied process based on the usage of organochlorosilanes [2, 5, 9]. The samples of MQ copolymers investigated in this paper were obtained by the hydrolytic polycondensation in the presence of an active medium [10]. The investigation of the reaction of organoalkoxysilanes with excess of acetic acid showed that the full conversion of alkoxide groups of multifunctional oligomers or their mixtures with alkoxysilanes can be achieved. An acetic acid in this case acts as an active medium of the reaction. In contrast to conventional organic solvents an active medium not only solves initial reagents and products generated in the reaction, but also acts as reagent itself. In particular, it was shown that the usage of the excess of the anhydrous acetic acid leads to a hydrolytic polycondensation in which the water required is generated in reacting system so the full conversion of alkoxy groups is achieved [10].

Silicon 29 solid-state nuclear magnetic resonance (NMR) has proved to be a powerful tool in determination of mono-, di-, tri-, and tetrafunctional cross-linked groups and various substituents in polysiloxanes [9, 12–14]. Cross-polarization (CP) and direct polarization (DP) excitation methods in concert with magic spinning (MAS) allows one to obtain qualitative and quantitative information on composition, surface and bulk properties, local molecular environment of diverse silicon containing materials such as silica gels [12, 15], organosilicon polymers and resins [9, 16], mesoporous materials [17, 18], hybrid inorganic–organic materials [4, 5].

From this point of view the techniques mentioned above seems to be attractive for MQ copolymers investigations since it allows to conduct comprehensive analysis of material and to obtain information on composition and peculiarities of the molecular systems under examination.

Thus, the aim of the present paper is to probe a series of MQ copolymer samples by the solid-state NMR technique for the purpose of estimation of advantages of method for the investigation of complex hybrid systems such as MQ copolymers of various composition and practical application.

## Experimental

### MQ Copolymer

A mixture of tetraethoxysilane (20 g, 0.096 mol), trimethylmethoxysilane (10 g, 0.096 mol), and acetic acid (70 g, 1.167 mol) was boiled with a reflux condenser for 35 h, and toluene (100 mL) was added. The resulting mixture was washed with distilled water. After drying, filtration, and removal of solvents, a white solid was isolated with a yield of 84.1 % (11.4 g).

^1^H NMR (CDC1_3_): δ 0.13 (s, 9 H, (CH_3_)_3_Si) ppm.

Anal. calcd. (%): C, 25.505; H, 6.42; Si, 39.76.

Found (%): C, 24.72; H, 6.33; Si, 39.13.

The GPC curve shows a maximum at *M* = 3.5 × 10^3^.

Original polymer was divided into three different molecular weight fractions. Fraction with lowest molecular weight denoted as fraction 3, the highest molecular weight as fraction 1. Original polymer and fractions 1 and 2 appeared as white powders while fraction 3 appeared as a colorless resin-like substance. The GPC curves for fractions 1, 2, and 3 show maximum at 7.6 × 10^3^, 3.9 × 10^3^, and 2 × 10^3^, respectively.

### Solid-State NMR Experiments

Solid-state NMR experiments were performed on a Bruker Avance III spectrometer with a 9.4-T widebore superconducting magnet. The spectrometer operated at 400.22 MHz for ^1^H, 100.64 MHz for ^13^C, and 79.51 MHz for ^29^Si. Samples were placed in 3.2-mm rotor in diameter for spinning at magic angle. ^1^H and ^29^Si solution spectra were recorded in CDCl_3_ on a Bruker Avance III 500 MHz spectrometer operating at 500.2 and 99.38 MHz, respectively. All spectra were externally referenced to liquid tetramethylsilane (TMS) at 0 ppm. The experimental conditions are listed below using the following symbols: $\nu_{\text{R}}$ denotes sample rotation rate, $\nu_{\text{rf}}^{\text{H}}$ and $\nu_{\text{rf}}^{\text{X}}$ the magnitudes of radiofrequency magnetic fields applied to ^1^H and X spins, $\tau_{\text{CP}}$ the contact time, $\tau_{\text{RD}}$ the recycle delay, and NS the number of scans.

^29^Si: $\nu_{\text{R}}$ = 5 kHz, $\nu_{\text{rf}}^{\text{H}}$ during excitation = 100 kHz, during CP = 45 kHz, during SPINAL-64 decoupling = 50 kHz, $\nu_{\text{rf}}^{\text{Si}}$ = 45 kHz, $\tau_{\text{RD}}$ = 5–6 s for CP experiments, $\tau_{\text{RD}}$ = 250 s for DP, $\tau_{\text{CP}}$ = 10, NS = 1,024 and 256 for CP and DP experiments, respectively. For *T*_1_ measurements the set of 10 points with recovery times ranging from 0.1 to 120 s was applied.

^13^C: $\nu_{\text{R}}$ = 8 kHz, $\nu_{\text{rf}}^{\text{H}}$ during excitation and SPINAL-64 decoupling = 100 kHz, during CP $\nu_{\text{rf}}^{\text{H}}$ = 55.5 kHz, $\nu_{\text{rf}}^{\text{C}}$ during CP = 85 kHz, $\tau_{\text{CP}}$ = 1 ms, $\tau_{\text{RD}}$ = 5 s.

^29^Si solution state spectra were recorded using 30° pulse with 5 μs length.

## Results and Discussion

**Fig. 2:**
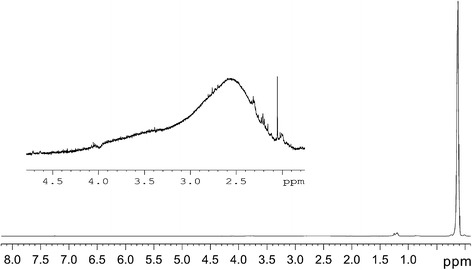
^1^H spectra of initial MQ copolymer in CDCl_3_ solution. In the insertion the spectrum in the region from 2 to 4.5 ppm scaled by 500/1 is shown

**Fig. 3:**
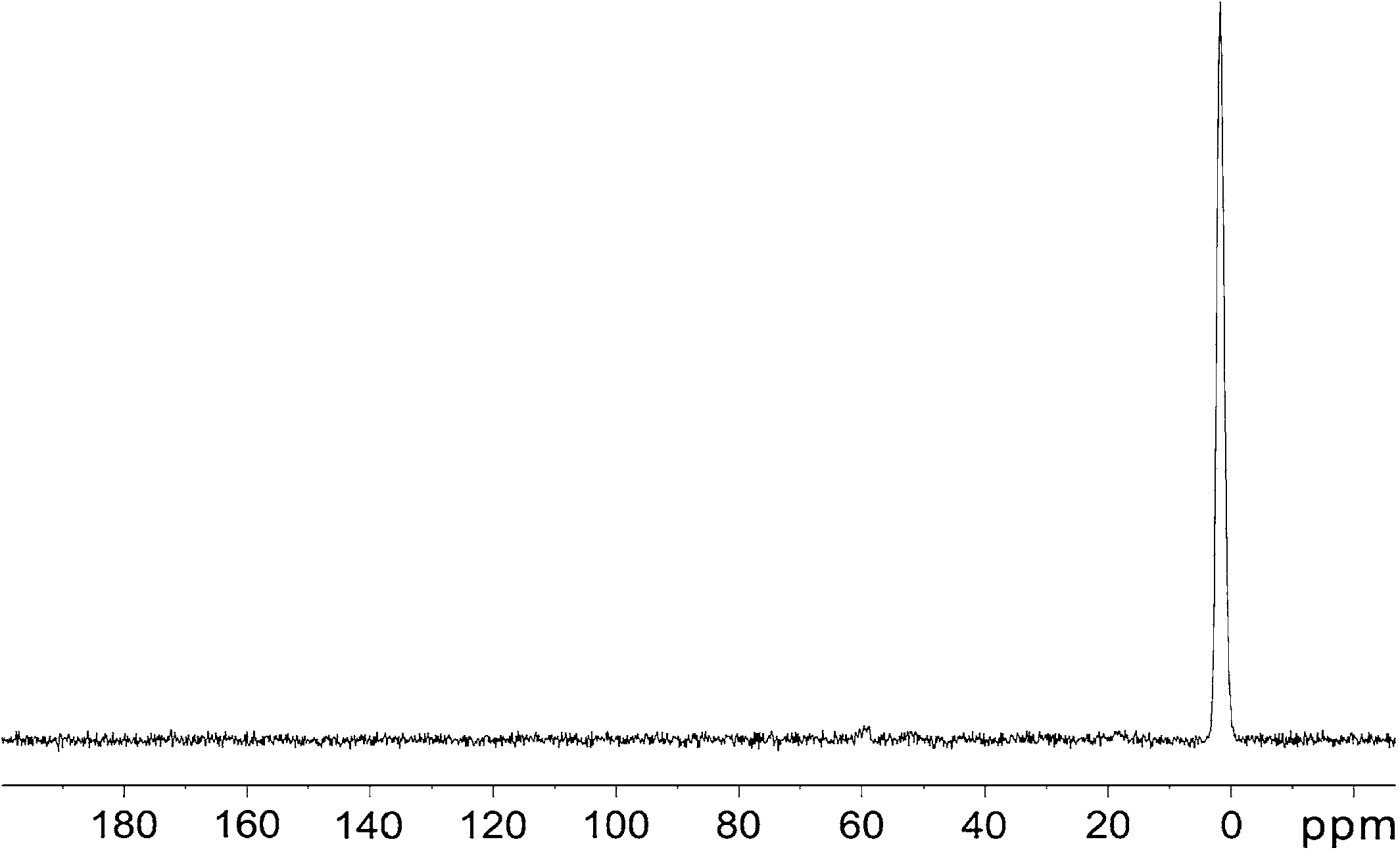
Solid state ^13^C MAS NMR spectrum

^1^H spectra of initial sample in CDCl_3_ solution and ^13^C spectra in solid state are shown in Figs. 2 and 3, respectively. The similar pattern was observed for fractions 1–3 of original polymer. ^1^H spectra reveal strong narrow CH_3_ signals at 0.13 ppm and very weak wide line at 2.6 ppm (Fig. 2). The wide line is attributed to the hydrogen atom of silanol groups (SiOH). The relative parts of silanol protons compared with methyl protons are 1/50 −1/40. ^13^C spectra in solution as well as MAS solid-state spectra show singlet line at 1.73 ppm, which belongs to CH_3_ groups (Fig. 3). Thus, ^13^C and ^1^H spectra confirm that organic part of molecule consists of CH_3_ groups which confirm the elemental analysis data.

Direct polarization ^29^Si spectra for the initial sample and two fractions extracted from it (Eqs. 1 and 2) were acquired under MAS at 5 kHz with 30° pulse. Spectrum of the fraction 3, which was a liquid-like, was recorded in CDCl_3_ solution. In this case the presence of background signals of probe in the region of Q resonances (between −80 and −120 ppm) was observed. Spectrum for background signals from the glass sample tube and the probe insert was recorded separately with the NMR tube filled with pure CDCl_3_ under the same experimental conditions and then was subtracted from the spectra of MQ copolymer. Under the spinning rate used in our experiments spectra for solid samples were analogous to the spectra obtained in solutions.

**Fig. 4:**
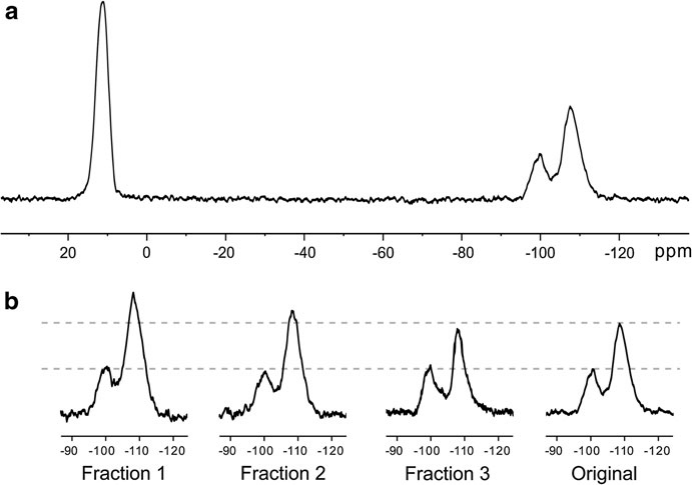
^29^Si NMR spectrum under 5 kHz MAS of the initial sample (**a**); the region of Q resonances for the original MQ copolymer and fractions obtained from it (**b**)

Three different resonances in ^29^Si spectra are observed: 12, −100, and −108 ppm (Fig. 4a). In general, ^29^Si NMR chemical shift show that an increase in the net positive charge of the silicon (Q^+^) causes an upfield shift in δ (i.e., more negative δ) [13]. Common notation for Q units also include superscript *n*, where *n* = 0–4 to indicate the number of Si atoms attached through the oxygen to the unit in question. Thus, Q^0^ denotes a silicon bonded through oxygen to no other network-forming elements, whereas Q^4^ denotes a silicon bonded through oxygen to four other silicons. The chemical shift becomes increasingly negative with each additional Si–O–Si linkage, due to increased electronic shielding of the central Si. Signal at 12 and −108 ppm were assigned to M (≡SiO *Si* (CH_3_)_3_) and Q^4^ ((≡SiO)_4_ *Si*) resonances, respectively, which is in a good agreement with the literature data [9, 15, 17]. Signal at −100 ppm is typically attributed to Q^3^ (*Si* (OSi)_3_OR) sites, where R denotes H or some hydrocarbon substituent [12, 13, 15–18]. The lower part of the Fig. 4 represents the region of Q resonances for the initial copolymer and fractions 1–3 (scales of the spectra chosen so that the M unit intensities are coincide).

**Fig. 5:**
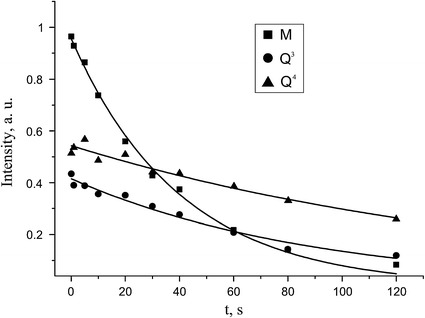
Longitudinal magnetization decay for initial MQ copolymer of distinct lines in ^29^Si spectrum. *Squares* correspond to experimental values of integral intensities of M sites, *circles* and *triangles* correspond to Q^3^ and Q^4^ units, respectively. The *lines* represent least square fits using Eq. (1)

NMR relaxation parameters were required for quantitative evaluation of silicon in different sites of initial MQ copolymer and its fractions. The ^29^Si spin–lattice relaxation times (*T*_1_) for the initial sample were measured by method proposed by Torchia [19]. Dependency of the signal intensities on longitudinal magnetization recovery time *t* in this case is given by

1 $$ M_{x} = M_{0} \exp \left( { - \frac{t}{{T_{1} }}} \right) , $$

where $M_{x}$ is the signal intensity, $M_{0}$ is the magnetization enhanced by CP. Results of experiment are presented in Fig. 5. The longitudinal relaxation times *T*_1_ are 40 ± 1 s for M unit, 90 ± 6 s for Q^3^ unit, 170 ± 15 s for Q^4^ unit. With these ratios between relaxation times and repetition delay $\tau_{\text{RD}}$ in the pulse sequence, according to expression [20]:

2 $$ M_{x} = M_{0} \sin \beta \frac{{1 - \exp ( - \tau_{\text{RD}} /T_{ 1} )}}{{1 - \exp ( - \tau_{\text{RD}} /T_{ 1} )\cos \beta }} , $$

where $M_{x}$ is the detected magnetization, $M_{0}$ is the equilibrium magnetization, *β* is the magnetization rotation angle under the action of pulse, the ratio of line intensities in the spectra (Fig. 4) remains unperturbed and could be used for quantitative analysis. Thus, for $\beta = 30^{ \circ }$, $\tau_{\text{RD}}$=250 s and *T*_1_ = 200 s (a time greater than the highest obtained in experiment) the value of fractional multiplier in Eq. (2) is ≈0.95. Thereby decrease in intensity caused by saturation for the line with highest relaxation time is <5 %, which obviously would be less for lines with shorter relaxation times. Relative concentrations of silicon in different groups were obtained by fitting lines in Fig. 4 by Gaussian line-shapes. Areas under lines were normalized by the area of M units. Corresponding results are listed in Table 1.

**Table 1:** Relative content of silicon in different units of MQ copolymer and its fractions obtained by DP ^29^Si NMR

	M	Q	Q^3^	Q^4^
Original	1	0.99 ± 0.05	0.4 ± 0.05	0.58 ± 0.05
Fraction 3	1	0.92 ± 0.05	0.32 ± 0.05	0.6 ± 0.05
Fraction 2	1	1.07 ± 0.05	0.28 ± 0.05	0.79 ± 0.05
Fraction 1	1	1.29 ± 0.05	0.38 ± 0.05	0.91 ± 0.05

Data represented in Table 2 show that the ratio between M and Q units in fractions changes in comparison to initial copolymer. The comparative part of Q (Q = Q^3^ + Q^4^) units grows with the molecular weight of the fraction. Repartition of the Q unit intensities resulted mostly by the increasing of the Q^4^ unit fraction. The amount of Q^3^ groups is about three per ten MQ unit. This value is 1.5–1.9 times higher compared with ^1^H NMR data.

**Table 2:** Parameters of CP dynamics obtained for initial MQ copolymer

	$M_{\text{CP}}$	$T_{1\rho }^{\text{H}}$, ms	$T_{\text{SiH}}$, ms
M	1	132 ± 5	0.9 ± 0.1
Q^3^	0.29	130 ± 5	0.8 ± 0.1
Q^4^	0.71	129 ± 5	1.3 ± 0.1

Diversity of *T*_1_ values for lines in different positions indicates the difference in mobility and chemical environment. Main mechanism determining relaxation rate of ^29^Si nuclei in our case is dipole–dipole interaction of ^29^Si with protons. For silicon nuclei of Q units *T*_1_ values are considerably higher than that obtained for M units. This fact evidences the greater remoteness of Q units from protons of methyl groups. The values for Q^4^ site are substantially higher than those obtained for Q^3^. The dominating relaxation mechanism for M and Q^4^ units is dipole–dipole interaction ^29^Si with methyl group’s protons. For Q^3^ unit the additional relaxation way due to ^29^Si silanol proton is also possible. Anyhow, the main ^29^Si relaxation path for Q^3^ is the dipole–dipole interaction between ^29^Si and methyl group protons by the reason of their high abundance in the sample and rapid rotating motion. Thereby silicon *T*_1_ values should reflect the distance between ^29^Si spin and the methyl group. For silicon nuclei of Q units *T*_1_ is considerably larger compared with *T*_1_ of M units. The spin–lattice relaxation value increases in the next order $T_{1}^{\text{Si}} ({\text{M}}) > T_{1}^{\text{Si}} ({\text{Q}}^{3} ) > T_{1}^{\text{Si}} ({\text{Q}}^{4} )$. Therefore, we may propose that MQ molecule consist of the core formed by Q^4^ units which is surrounded by the shell of Q^3^ and M units.

**Fig. 6:**
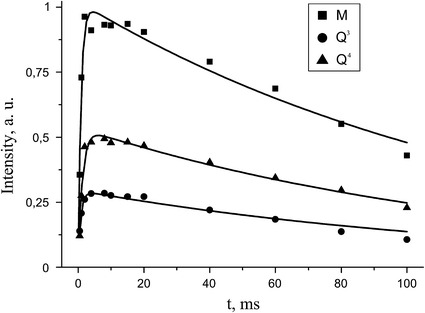
Plots of peak heights in ^29^Si CP/MAS NMR spectra as a function of the contact time for initial MQ copolymer. The *squares* correspond to M units, and the *circles* and *triangles* correspond to Q^3^ and Q^4^ units, respectively. The *lines* represent least square fits using Eq. (3)

The direct polarization is the straightest way to obtain quantitative spectra, but this technique is time-consuming method especially in the case of ^29^Si nuclei. By the reason of long repetition time requirements for quantitative analysis the only limited number of scans could be acquired in acceptable time of experiment, so DP spectra have low signal to noise ratio which affects the resulting accuracy. Cross-polarization allows to obtain the more intense signal with respect to DP spectra (the CP gain of signal is proportional to the ratio of gyromagnetic ratios of nuclei pair, which in the case of ^29^Si and ^1^H is about 5) in the more shorter time (the required repetition time depends on $T_{1}^{\text{H}}$ rather than $T_{1}^{\text{Si}}$). However, one should take into account that intensities of the lines in CP spectra are determined not only by the number ^29^Si nuclei but also by the cross-polarization dynamics. The CP dynamics are affected by changes in molecular structure and the number of ^1^H sites in close proximity to ^29^Si site as well as by any molecular motion in the system. To investigate the CP dynamics the signal intensities were measured as a function of the contact time for initial sample of MQ copolymer (Fig. 6).

The dependences were analyzed using the following Eq. [21]:

3 $$ M(t) = M_{\text{CP}} \left[ {\exp \left( { - \frac{t}{{T_{1\rho }^{\text{H}} }}} \right) - \exp \left( { - \frac{t}{{T_{\text{SiH}} }}} \right)} \right]\left( {1 - \frac{{T_{\text{SiH}} }}{{T_{1\rho }^{\text{H}} }}} \right)^{ - 1} , $$

where $M(t)$ is the signal intensity, $M_{\text{CP}}$ is magnetization enhanced by CP, $T_{1\rho }^{\text{H}}$ is the ^1^H spin–lattice relaxation time in the rotating frame, and $T_{\text{SiH}}$ is the cross-relaxation time between the ^29^Si and ^1^H spins. Each experimental curve is fitted to Eq. (3) by adjusting $M_{\text{CP}}$, $T_{1\rho }^{\text{H}}$, and $T_{\text{SiH}}$. The obtained values are listed in Table 2.

$T_{1\rho }^{\text{H}}$ values are the same for all three lines in the spectra. This confirms that the main source of relaxation of silicon in different units of copolymer is methyl group protons. Silicon of M groups is in the closest distance to CH_3_ groups compared with the neighboring Q^3^ and Q^4^ units. It is expected that this group should posses the least $T_{\text{SiH}}$ values. The values of $T_{\text{SiH}}$ depends on the magnitude of the ^29^Si–^1^H dipolar coupling, which is related to the distance between ^29^Si and ^1^H spins. As it is shown in Table 2 the $T_{\text{SiH}}$ values are the same for M and Q^3^ units and slightly larger for Q^4^. This fact let us to assume that Q^3^ units are located at the surface. Therefore, it may be concluded that MQ copolymers possess the compact structure with core composed of Q^4^ units surrounded by Q^3^ and M units forming a shell of the particle. This result agrees well with spin–lattice relaxation data.

**Fig. 7:**
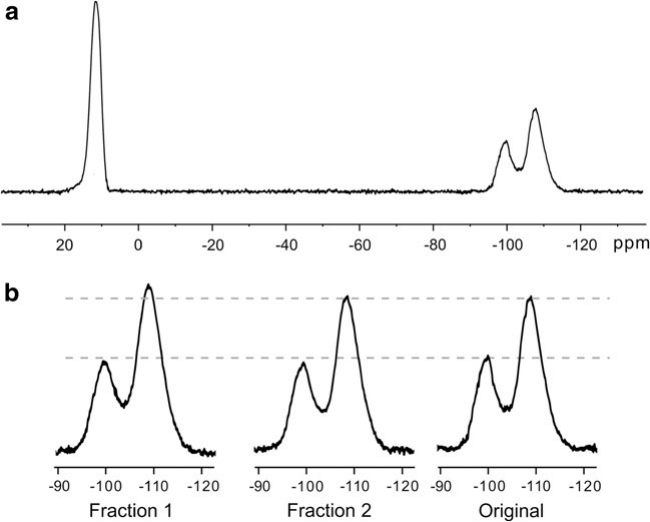
^29^Si NMR spectra under 5 kHz MAS of the original MQ copolymer using CP with 10 ms contact duration (**a**); the region of Q resonances for the original MQ copolymer and fractions obtained from it (**b**)

In Fig. 7 the CP spectra recorded at the contact time equal to 10 ms are shown. Spectra were decomposed in individual Gaussian components to obtain peak area. The values of signal areas were corrected according to Eq. (3) with values of $T_{\text{SiH}}$ and $T_{1\rho }^{\text{H}}$ from Table 2. Corresponding data on distinct groups content for fractions 1 and 2 and initial sample are presented in Table 3. Fraction 3 was unsuitable for such experiment as it appeared as liquid-like substance.

**Table 3:** Relative concentration of silicon in different units of initial MQ copolymer and fractions obtained by ^1^H-^29^Si CP/MAS NMR

	M	Q	Q^3^	Q^4^
Original	1	1.03 ± 0.05	0.34 ± 0.05	0.69 ± 0.05
Fraction 3	–	–	–	–
Fraction 2	1	1.01 ± 0.05	0.32 ± 0.05	0.69 ± 0.05
Fraction 1	1	1.09 ± 0.05	0.34 ± 0.05	0.75 ± 0.05

Amount of Q^4^ units increases as the molecular weight of fraction increased (Table 3). Percentage of Q^4^ lower than that obtained in DP experiment but the main tendency of increasing of Q groups is conserved. Data also shows that content of Q^3^ in relation to M units remain constant among fractions.

## Conclusions

Silicon-29 NMR measurements provide valuable information in characterizing of structure of MQ copolymers. Particularly, ratio of M/Q units in copolymer is determined. This ratio is close to the unity which is close to the ratio determined by initial reaction conditions, where the ratio of monomers forming M (trimethylmethoxysilane) and Q (tetraethoxy) units are also equal. The results obtained for fractions of initial copolymer shows increasing in Q units content with the increasing of molecular weight of the fraction. On the whole, the obtained data confirm that the synthesized MQ copolymer consist of densely linked silica core consisting of Q^4^ ((≡SiO)_4_ *Si*) units surrounded by the shell of M (≡SiO *Si* (CH_3_)_3_) units.
